# Prevailing Food Safety and Sustainability Practices in Grocerants: A Cross‐Sectional Study in Three Countries

**DOI:** 10.1155/ijfo/9949256

**Published:** 2026-04-25

**Authors:** Surya Sasikumar Nair, Aparna Porumpathuparamban Murali, Richard Atinpoore Atuna, Fortune Akabanda, Wojciech Kolanowski, Shoukui He, Joanna Trafiałek

**Affiliations:** ^1^ Department of Food Gastronomy and Food Hygiene, Institute of Human Nutrition Sciences, Warsaw University of Life Sciences, Warsaw, Poland, sggw.pl; ^2^ Department of Food Science and Technology, University for Development Studies, Tamale, Ghana, uds.edu.gh; ^3^ Health Care Institute, State University of Applied Sciences in Przemyśl, Przemysl, Poland; ^4^ School of Agriculture and Biology, Shanghai Jiao Tong University, Shanghai, China, sjtu.edu.cn

**Keywords:** compliance, food safety, food system, grocerants, restaurant, retail, sustainability

## Abstract

Ensuring food safety while advancing sustainability is increasingly recognised as a dual imperative for modern food systems. However, the interconnection between food safety and sustainability at the retail and food service levels remains underexplored. This study is aimed at assessing and comparing the prevailing food safety practices (FSP) and sustainability practices (SUST) among grocerants, which are hybrid outlets combining grocery retail and restaurant functions, in Ghana, Poland, and India. A structured 28‐indicator observational checklist (14 FSP and 14 SUST indicators) was applied during unannounced audits of 150 grocerants. The analysis revealed significant cross‐country differences in compliance levels for both sections. Polish grocerants achieved comparatively the highest mean scores, reflecting advanced hygiene management and proactive SUST, whereas Ghanaian and Indian outlets exhibited moderate compliance. Cluster analysis identified three groups representing low‐, transitional‐, and high‐maturity stages of sustainable food safety performance. Grocerants with stronger food safety compliance also demonstrated higher sustainability engagement, suggesting an association between the two domains. The findings highlight differences in regulatory enforcement and managerial commitment across various contexts, contributing to the development of the concept of sustainable food safety management, which links hygiene compliance with environmental and social responsibility in emerging retail formats.

## 1. Introduction

According to FAO [1], a sustainable food system ensures food security and nutrition for all while preserving economic, social, and environmental foundations for future generations. It must be economically viable, socially beneficial, and environmentally sustainable, aligning with the United Nations′ Sustainable Development Goals (SDGs), contributing directly to at least 12 of the 17 SDGs [1, 2]. Achieving sustainability in the food system requires coordinated efforts across the entire food chain, from production and processing to storage, distribution, retail, and waste management, addressing both individual and population‐level health outcomes [3].

Most research on sustainability in the food sector has focused on its relationship with food security, emphasizing availability, accessibility, and environmental impact [3–5]. However, the interconnection between food safety and sustainability, though equally essential, has received limited attention. Safe food is a nonnegotiable element of a sustainable food system, as unsafe food leads to waste, economic loss, and public health risks, thereby undermining environmental and social sustainability [6]. Conventional food safety management systems (FSMS), such as HACCP and ISO standards, primarily focus on hazard control while largely overlooking environmental sustainability dimensions, thereby calling for integrated and “green” approaches to food safety management [7].

Within this context, food businesses are increasingly expected to assess the impact of their operations on society and the environment and to adopt verifiable sustainable practices. However, there are no established indicators or reporting frameworks to evaluate sustainability performance for many sectors, including retail operations [2, 8]. Retail environments, particularly grocerants, serve as a critical interface where food safety and sustainability intersect. The term “grocerant,” a portmanteau word that combines the words “grocery” and “restaurant,” describes hybrid outlets offering freshly prepared, ready‐to‐eat foods within retail settings such as supermarkets, convenience shops, and chain drug shops [9]. These establishments embody the modern consumer demand for convenience and freshness while posing new challenges for integrating safe food handling with sustainable operational practices [10].

Despite the growing awareness of sustainability in the global food industry, empirical research that jointly examines food safety and sustainability practices (SUST) at the retail and food service levels remains scarce. Most available research has studied either FSMS or environmental sustainability programs in isolation [11–14]. Although recent conceptual models, such as Green HACCP, have been proposed to integrate environmental and food safety objectives, there remains a lack of empirical studies validating these concepts [7]. Furthermore, existing research primarily originates from developed countries, leaving a significant gap in understanding how enterprises across diverse regulatory and economic contexts address these two imperatives. Retail and food service enterprises are facing increasing challenges in ensuring food safety, particularly as prepared food sales increase in grocery and convenience stores. Such trends highlight the dual responsibility of maintaining rigorous hygiene standards while minimizing the environmental footprint of daily operations [15, 16]. Grocerants, hybrid outlets that combine grocery retail and food service activities [17], provide an ideal context for evaluating these dynamics, since their operational model necessitates adherence to stringent food safety requirements alongside resource efficiency and environmental responsibility. Understanding how grocerants perform in different governance frameworks can reveal differences in compliance and assess the development of sustainable food safety practices (FSP) across national systems.

Against this backdrop, the present study is aimed at assessing and comparing the prevailing FSP and sustainable practices (SUST) among grocerants in Ghana, Poland, and India. The following hypotheses were formulated:

H1: There are significant differences in the level of compliance with FSP and SUST among grocerants in Ghana, Poland, and India.

H2: There are significant relationships between FSP and SUST among grocerants.

The corresponding null hypotheses were formulated as follows:

H0_1_: There are no significant differences in the level of compliance with FSP and SUST among grocerants in Ghana, Poland, and India.

H0_2_: There are no significant relationships between FSP and SUST among grocerants.

By providing cross‐country insights into how grocerants implement these intertwined dimensions, this study contributes to advancing the concept of sustainable food safety management. This growing perspective links hygiene compliance, environmental sustainability, and social accountability in the retail food service sector.

## 2. Materials and Methods

### 2.1. Study Design and Data Collection

This study employed a cross‐sectional design to assess the prevailing food safety and SUST among grocerants in three countries. The study was conducted in three countries: Ghana (Africa), Poland (Europe), and India (Asia), representing three continents. These countries were selected because they represent different food safety governance systems and sustainability approaches within transitional, developed, and developing economies. Ghana was chosen as a representative of the sub‐Saharan African context, Poland as a European Union (EU) member with harmonized food safety standards, and India as a rapidly developing Asian country with evolving retail and regulatory systems.

A direct observation methodology was employed to systematically evaluate FSP and SUST. In the present study, we designed a structured inspection checklist and used it for direct observation in shops in Ghana, Poland, and India, using Google Forms. The primary objective of the inspection checklist was to assess and compare compliance levels with food safety and SUST among Grocerants (retail food service shops) across Ghana, Poland, and India. To ensure objectivity and accuracy, the on‐site observational audit was conducted by a qualified inspector without prior announcement to the grocerants. The inspections were performed while the food shop employees were engaged in their regular work activities, allowing for an authentic assessment of the actual practices. The inspection checklist was completed through direct observation without any interaction with food handlers.

The inspection checklist was made up of 28 questions (Table 1), which had been validated through a pilot study by the research authors for understandability, consistency, and adequacy [12, 18]. The indicators were grouped into two sections. The first section focused on assessing FSP implemented within retail shops, capturing aspects such as hygiene, handling, and compliance with food safety management practices. The second section addressed SUST, emphasizing environmentally responsible operations, waste management, and resource‐efficient activities adopted in the retail environment.

**Table 1:** The inspection checklist used in the study.

Q. no.	Scope	Abbreviation
Section: Food safety practices		FSP
Q1	Shop layout supports hygienic food handling.	Layout
Q2	Adequate ventilation and lighting to maintain safe food handling conditions.	Ventilation and lighting
Q3	Food safety information (manuals, posters, signs, or icons) is clearly displayed in the shop.	Food safety information
Q4	Food preparation areas are maintained in a sanitary condition.	Sanitary area
Q5	Products are stored under correct conditions (e.g., refrigerated, frozen, or hot held).	Storage
Q6	High‐risk items such as raw meat or seafood are kept separate from direct customer contact.	High‐risk separation
Q7	Expired products are not displayed.	Expiry control
Q8	Displayed foods are protected from customer handling or contamination.	Display protection
Q9	Employees wear appropriate protective clothing while handling food.	Protective wear
Q10	Employees follow hygienic practices when serving or preparing food.	Hygiene behavior
Q11	Food contact surfaces, equipment, and utensils are visibly clean.	Clean surface
Q12	Cleaning and sanitizing routines are carried out regularly.	Cleaning routine
Q13	There are no signs of pest infestation inside the shop.	Pest signs
Q14	Pest control devices are present and maintained in working order.	Pest devices
Section: Sustainability practices		SUST
Q15	Alternative sustainable packaging options	Alternative packaging
Q16	Energy‐efficient appliances and equipment	Energy‐efficient appliances
Q17	Eco‐friendly cleaning products	Eco‐clean
Q18	Discounts on surplus food/minimizing food waste	Discounts
Q19	Food donations to shelters/charities	Food donation
Q20	Store charges for single‐use bags	Bag charge
Q21	Waste segregation and recycling systems	Waste segregation
Q22	Prohibiting noncompostable labels on fruit or vegetables	Label prohibition
Q23	Sustainable refrigeration systems	Sustainable refrigeration
Q24	Use of natural light in design	Natural light
Q25	Organic fruits and vegetables are available.	Organic products
Q26	Store accepts private food containers and bags	Private bag
Q27	Zero plastic packaging	Zero plastic
Q28	Local products are dominant in the store.	Local products

An ordinal rating scale was used for assessing the level of compliance with each indicator. The highest score awarded was 4, corresponding to full compliance, as all requirements were met. A score of 3 indicates partial compliance, which is a slight deviation from full compliance. A score of 2 indicates partial compliance with only limited efforts observed, whereas a score of 1 indicates noncompliance. Higher scores denote better compliance with respective practices. The scoring system and scale used were modified from the study by [12]. Data collection was predominantly carried out through shop observations of 150 randomly selected grocerants (with an equal number from each country) from January to May 2024.

### 2.2. Statistical Analysis

As the scale data were not normally distributed (Shapiro–Wilk test), nonparametric statistical tests were applied. The internal consistency of the inspection checklist sections was evaluated using Cronbach′s alpha. Descriptive statistics were performed to determine the mean scores and standard deviation for determining the prevailing compliance levels across outlets. The Kruskal–Wallis test (post hoc: multiple pairwise comparisons) was performed to find the statistical differences between each of the questions and countries [19]. A hierarchical cluster analysis was performed to determine how the shops were grouped based on the current compliance with FSP and SUST. Ward′s method and the squared Euclidean distance method were applied for clustering. Significances between the mean scores for the FSP and SUST sections across the three clusters were analyzed using the nonparametric Kruskal–Wallis test, with the importance of results established at *p* < 0.05 [12]. The relationship between FSP and SUST practices in grocerants was evaluated using Spearman′s rank‐order test. All statistical analyses were conducted using Statistica Version 13.3 (TIBCO Software Inc., Palo Alto, California, United States).

## 3. Results

The reliability of the inspection checklist was assessed using Cronbach′s alpha test to determine the internal consistency of the grouped items [20]. The FSP section (Q1–Q14) demonstrated excellent reliability, with an *α* coefficient of 0.920. In contrast, the sustainable practices (SUST) section (Q15–Q28) yielded a coefficient of *α* = 0.872, indicating high reliability of the instrument.

**Figure 1:**
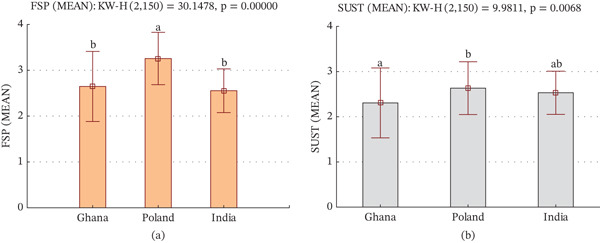
Box plots (±SD) showing differences in prevailing (a) food safety practices (FSP) scores (Likert scale 1–4) and (b) sustainability practices (SUST) scores across Ghana, Poland, and India. Kruskal–Wallis tests indicated significant differences (*p* < 0.001). Different letters above the bars denote significant pairwise differences between countries based on post hoc test multiple comparisons of mean ranks (*p* < 0.05).

Figure 1 presents the mean compliance scores for the FSP and SUST sections across the three countries. Based on the Kruskal–Wallis test, significant differences were observed in compliance levels among countries for both FSP (*χ* ^2^ = 30.1478, *p* < 0.05) and sustainable practices (SUST; *χ* ^2^ = 9.981, *p* = 0.0068). These results confirm that compliance levels vary across national contexts. Post hoc multiple comparisons revealed that Polish grocerants had considerably higher FSP scores than those in Ghana and India (*p* < 0.05), but there was no significant difference between Ghana and India (Figure 1a). Likewise, for the SUST section, post hoc analysis confirmed that Polish grocerants outperformed in Ghana (*p* < 0.05), whereas India did not significantly differ from either group (see also Figure 1b).

### 3.1. Prevailing FSP Compliance

To provide an overview of the prevailing FSP across each country, Table 2 depicts mean scores (±SD) for the assessed indicators used to determine the prevailing FSP of the grocerants. Table 2 shows Polish grocerants exhibited the highest overall compliance, with mean scores predominantly between 3.0 and 3.7, corresponding to partial to full compliance across most indicators. The highest scores were recorded for Q6 (high‐risk items such as raw meat or seafood are kept separate from direct customer contact), Q7 (expired products are not displayed), and Q8 (displayed foods are protected from customer handling or contamination). These findings indicate strong implementation of preventive hygiene measures and systematic monitoring of product segregation and display conditions. However, a comparatively lower adherence was noted for Q14 (pest control devices are present and maintained in working order), suggesting that pest management systems may not be as rigorously maintained or regularly inspected as other hygiene practices.

**Table 2:** Mean ± SD scores of food safety practices (FSP) across Ghana, Poland, and India.

Q. no.	Questions	Ghana	Poland	India	KW‐H test^*^
Q1	Layout	3.1 ± 0.93^a^	2.8 ± 0.99^a^	2.2 ± 0.82^b^	18.00, *p* < 0.001
Q2	Ventilation and lighting	3.0 ± 1.05	3.0 ± 0.90	2.6 ± 0.93	5.57, *p* = 0.061
Q3	Food safety information	2.1 ± 1.22^a^	3.0 ± 1.02^b^	1.9 ± 0.77^a^	25.98, *p* < 0.001
Q4	Sanitary area	3.2 ± 0.82^a^	3.3 ± 0.80^a^	2.8 ± 0.76^b^	13.57, *p* = 0.001
Q5	Storage	2.6 ± 1.05^a^	3.6 ± 0.70^b^	2.6 ± 0.88^a^	32.43, *p* < 0.001
Q6	High‐risk separation	2.5 ± 1.07^a^	3.7 ± 0.61^b^	3.0 ± 0.70^a^	39.19, *p* < 0.001
Q7	Expiry control	2.2 ± 0.95^c^	3.7 ± 0.67^a^	3.2 ± 0.74^b^	58.49, *p* < 0.001
Q8	Display protection	2.5 ± 0.97^b^	3.7 ± 0.54^a^	3.0 ± 0.80^b^	42.04, *p* < 0.001
Q9	Protective wear	2.3 ± 1.23^b^	3.0 ± 1.12^a^	2.0 ± 0.92^b^	19.43, *p* < 0.001
Q10	Hygiene behavior	3.2 ± 0.78	3.2 ± 0.77	3.1 ± 0.97	0.34, *p* = 0.84
Q11	Clean surface	2.8 ± 0.93^b^	3.6 ± 0.70^a^	2.8 ± 0.62^b^	32.41, *p* < 0.001
Q12	Cleaning routine	2.9 ± 0.84^a^	3.0 ± 0.99^a^	2.4 ± 0.85^b^	14.36, *p* = 0.001
Q13	Pest signs	2.4 ± 0.99^b^	3.4 ± 0.76^a^	2.2 ± 0.81^b^	43.00, *p* < 0.001
Q14	Pest devices	2.2 ± 1.27^ab^	2.6 ± 1.01^a^	2.0 ± 0.95^b^	7.26, *p* = 0.027

*Note:* Different letters above the bars denote significant pairwise differences between countries based on post hoc test multiple comparisons of mean ranks (*p* < 0.05). No letter denotes no significance.

^*^Kruskal–Wallis tests indicated significant differences (*p* < 0.001).

Ghanaian and Indian grocerants generally showed moderate compliance with FSP, with mean scores ranging from 2.1 to 3.2, indicating partial fulfillment of most requirements with consistent effort. As shown in Table 2, for Ghana, the highest levels of adherence were observed for food preparation areas maintained in a sanitary condition (Q4, 3.2 ± 0.8), shop layout supporting hygienic food handling (Q1, 3.1 ± 0.9), and employees following hygienic practices during food preparation (Q10, 3.2 ± 0.8). These findings suggest that Ghanaian establishments prioritised cleanliness and staff hygiene behavior. However, relatively lower compliance was noted for display of food safety information (Q3, 2.1 ± 1.2) and management of expired products (Q7, 2.2 ± 0.9), implying inconsistencies in documentation and product monitoring. For India, grocerants obtained relatively higher scores for keeping high‐risk items separate from direct customer contact (Q6, 3.0 ± 0.8), ensuring that expired products were not displayed (Q7, 3.2 ± 0.7), and protecting displayed foods from contamination (Q8, 3.0 ± 0.8). These results indicate that Indian establishments maintained satisfactory control over product display and the prevention of cross‐contamination. However, lower scores were recorded for the display of food safety information (Q3, 1.9 ± 0.9) and pest control measures (Q14, 2.0 ± 0.9), suggesting limited communication of food safety instructions and inadequate pest control maintenance.

Kruskal–Wallis test (post hoc: multiple comparisons of mean ranks) was performed on the mean scores of FSP variables per country to assess whether differences shown in Table 2 were statistically significant. The study found significant cross‐country differences in shop layout design, food safety information display, high‐risk item separation, and food protection practices among Polish, Ghanaian, and Indian grocerants. Polish grocerants consistently achieved higher mean ranks, whereas Ghanaian and Indian grocerants displayed moderate adherence. No significant differences were found in ventilation, lighting, hygienic food preparation, or pest control devices.

### 3.2. Prevailing Sustainability Practice Compliance

Table 3 shows the mean scores used to assess the sustainability approaches of the grocerants. A partial level of compliance was observed among the three countries, with Ghanaian grocerants being lower than those in India and Poland. Ghanaian grocerants demonstrated moderate compliance with SUST, with mean scores between 1.6 and 2.7, indicating that most requirements were only partially met with limited to minimal efforts. As shown in Table 3, relatively better compliance was observed for energy‐efficient appliances and equipment (Q16, 2.7 ± 0.9), use of eco‐friendly cleaning products (Q17, 2.7 ± 1.0), and promotion of local products (Q28, 2.7 ± 0.9), suggesting that Ghanaian grocerants made moderate efforts to incorporate energy conservation and local sourcing into daily operations. However, lower mean scores were recorded for discounting surplus food to minimize waste (Q18, 2.1 ± 1.1), donation of surplus food to shelters or charities (Q19, 2.1 ± 1.1), charging for single‐use bags (Q20, 1.6 ± 0.9), reflecting limited or inconsistent waste‐reduction efforts, and waste segregation and recycling systems (Q21, 1.6 ± 0.9), indicating underdeveloped practices in packaging sustainability and waste management.

**Table 3:** Mean ± SD scores of sustainability practices (SUST) across Ghana, Poland, and India.

Q. no.	Questions	Ghana	Poland	India	KW‐H test^*^
Q15	Alternative packaging	2.4 ± 1.05^a^	3.1 ± 0.90^b^	2.6 ± 0.96^ab^	12.41, *p* = 0.002
Q16	Energy‐efficient appliances	2.7 ± 0.89^b^	3.2 ± 1.01^a^	2.6 ± 0.86^b^	13.93, *p* = 0.0009
Q17	Eco‐clean	2.7 ± 1.00^b^	3.3 ± 0.95^a^	2.6 ± 0.86^b^	17.46, *p* = 0.0002
Q18	Discounts	2.1 ± 1.09^ab^	1.7 ± 0.95^b^	2.4 ± 0.91^a^	14.84, *p* = 0.0006
Q19	Food donation	2.1 ± 1.15^a^	1.1 ± 0.48^b^	1.9 ± 0.81^a^	35.83, *p* < 0.001
Q20	Bag charge	1.6 ± 0.97^b^	3.4 ± 1.05^a^	3.0 ± 0.94^a^	57.86, *p* < 0.001
Q21	Waste segregation	1.6 ± 0.92^ab^	2.1 ± 1.20^a^	1.5 ± 0.91^b^	8.13, *p* = 0.017
Q22	Label prohibition	2.2 ± 1.14^b^	3.0 ± 1.07^a^	2.5 ± 0.79^b^	16.11, *p* = 0.0003
Q23	Sustainable refrigeration	2.5 ± 0.93	2.5 ± 1.40	2.2 ± 0.91	2.30, *p* = 0.317
Q24	Natural light	2.7 ± 1.00	2.7 ± 1.00	2.7 ± 0.96	0.32, *p* = 0.85
Q25	Organic products	2.5 ± 0.91^a^	2.0 ± 1.12^b^	2.5 ± 0.86^a^	11.42, *p* = 0.003
Q26	Private bag	2.0 ± 1.02^b^	3.8 ± 0.71^a^	3.7 ± 0.61^a^	79.40, *p* < 0.001
Q27	Zero plastic	2.5 ± 0.99	2.2 ± 0.97	2.3 ± 1.04	2.32, *p* = 0.31
Q28	Local products	2.7 ± 0.91	2.7 ± 1.05	2.9 ± 0.78	1.00, *p* = 0.607

*Note:* Different letters above the values denote significant pairwise differences between countries based on post hoc multiple comparisons of mean ranks (*p* < 0.05). No letter denotes no significance.

^*^Kruskal‐Wallis tests indicated significant differences (*p* < 0.001).

Polish grocerants exhibited comparatively high compliance with SUST, with mean scores ranging from 1.1 to 3.8, reflecting minimal to full compliance across parameters. The highest adherence was observed for accepting private food containers and bags (Q26, 3.8 ± 0.7), store charges for single‐use bags (Q20, 3.4 ± 1.0), and the use of eco‐friendly cleaning products (Q17, 3.3 ± 1.0). However, low compliance was noted for discounts on surplus food (Q18, 1.7 ± 0.9) and food donations shelters (Q19, 1.1 ± 0.5), showing limited adoption of social sustainability measures. Indian grocerants showed moderate compliance with SUST, with mean scores ranging from 1.5 to 3.7, indicating partial to full compliance across most parameters. Higher compliance was observed for acceptance of private food containers and bags (Q26, 3.7 ± 0.6), alternative sustainable packaging (Q15, 2.6 ± 0.9), eco‐friendly cleaning products (Q17, 2.6 ± 0.9), and use of energy‐efficient appliances (Q16, 2.6 ± 0.9). However, lower compliance was found for the prohibition of noncompostable labels (Q22, 2.5 ± 0.8), organic fruits and vegetables availability (Q25, 2.5 ± 0.9), and waste segregation systems (Q21, 1.5 ± 0.9), indicating inconsistent implementation of waste and material management practices.

The Kruskal–Wallis test confirmed that there were significant differences (*p* < 0.05) among Ghana, Poland, and India for selected sustainability indicators. Overall, SUST varied moderately among the countries, with no single region showing consistently higher compliance across all indicators. Significant variation was observed for indicators such as alternative packaging (Q15), energy‐efficient appliances (Q16), and eco‐friendly cleaning products (Q17). However, for other indicators such as use of natural lighting (Q24), zero plastic packaging (Q27), and promotion of local products (Q28), no significant differences (*p* > 0.05) were found, suggesting similar adoption levels across all three countries.

### 3.3. Hierarchical Cluster Analysis

A hierarchical cluster analysis was performed to determine how the grocerants grouped according to their prevailing compliance with FSP and SUST, based on the individual scores of 28 variables. Three clusters (A, B, and C) were obtained: Cluster A mainly comprised grocerants from Ghana (*n* = 26) with a few from Poland (*n* = 3); Cluster B included a mix of grocerants from Ghana (*n* = 13), Poland (*n* = 17), and a majority from India (*n* = 48); whereas Cluster C consisted predominantly of Polish grocerants (*n* = 30) along with Ghanaian (*n* = 11) and Indian (*n* = 2) grocerants. The resulting three clusters were further compared using mean FSP and SUST scores to evaluate significant differences among countries (Table 4).

**Table 4:** Mode scores, mean scores, and the significant differences for the prevailing food safety practices (FSP) and sustainability practices (SUST) among countries within Clusters A, B, and C.

Section	Country	Cluster A (*n* = 29)		KW‐H test	Cluster B (*n* = 78)		KW‐H test	Cluster C (*n* = 43)		KW‐H test
		Mode	Mean		Mode	Mean		Mode	Mean	
FSP	Ghana	2	2.5	0.00	2	2.4	4.25	4	3.9	1.52
	Poland	3	2.7	*p* = 1.00	3	2.8	*p* = 0.11	4	3.8	*p* = 0.46
	India	—	—	(*ns*)	3	2.6	(*ns*)	4	4.0	(*ns*)
SUST	Ghana	2	1.7	0.00	3	2.9	5.99	3	3.1	6.27
	Poland	1	1.0	*p* = 1.00	2	2.5	*p* = 0.05	3	2.9	*p* = 0.04
	India	—	—	(*ns*)	2	2.5	(**)	4	4.0	(*)

*Note:* Mode and mean values represent each country′s average compliance within clusters. Asterisk (*) symbolises significant differences between countries based on the Kruskal–Wallis nonparametric test (*p* < 0.05), whereas double asterisks (**) and (*ns*) denote borderline and no significance, respectively.

The Kruskal–Wallis test revealed no significant differences in FSP scores among countries within the clusters (*p* > 0.05). In contrast, SUST scores showed a borderline significant difference in Cluster B (*H* = 5.99, *p* = 0.05) and a significant difference in Cluster C (*H* = 6.28, *p* = 0.043), where Indian grocerants achieved higher compliance levels compared with Ghanaian and Polish counterparts. However, post hoc pairwise comparisons did not reveal clear differences between countries.

Cluster A represented grocerants with low overall compliance, characterised by minimal effort toward implementation, with mean FSP and SUST scores of around 2.5 and 1.7, respectively. Most of these shops were located in Ghana, indicating limited adherence to food safety and sustainability requirements. Cluster B reflected partial compliance, with mean scores between 2.6 and 2.8 for both FSP and SUST, and included a mixed distribution of shops from all three countries. In contrast, Cluster C consisted primarily of Polish and Indian grocerants and demonstrated higher compliance with both FSP (mean = 3.9–4.0) and SUST (mean = 3.1–4.0), representing the most advanced group in terms of sustainable food safety performance.

### 3.4. Association Between Food Safety and SUST in Grocerants

Table 5 presents the country‐wise Spearman association between food safety and SUST in grocerants. A statistically significant positive association was observed in Poland (*ρ* = 0.516, *p* < 0.001), indicating a stronger association between food safety management and SUST. In Ghana, a significant positive association was also identified (*ρ* = 0.357, *p* = 0.0109), suggesting an emerging alignment of these practices. Conversely, in India, the association was weak and not statistically significant (*ρ* = 0.172, *p* > 0.05).

**Table 5:** Country‐wise association between food safety and sustainability practices in grocerants.

Country	Spearman′s R (FSP/SUST)	*p*value
Ghana	0.357	0.011
Poland	0.516	< 0.001
India	0.172	0.231

## 4. Discussion

This study provides a cross‐sectional comparison of prevailing compliance levels in grocerants, assessing both FSP and SUST across countries with varying regulatory and governance systems. The results supported the first hypothesis, indicating statistically significant differences in the level of compliance with FSP and SUST among Ghana, Poland, and India. Meanwhile, the second hypothesis was partially supported, as statistically significant associations between FSP and SUST were found in Poland and Ghana but not in India. This underscores how varying regulatory environments and market maturity shape operational performance in blended retail food service settings. Based on the observation of FSP and SUST practices, grocerants across all three countries followed FSP as well as SUST with different compliance levels of approaches, suggesting that enterprises may possess distinct operational traits independent of national culture [12, 21].

Findings for Ghana demonstrated that FSMS are at a developing stage for the grocerants in our study, which probably explains the overall partial compliance level for the FSP indicators. Ghanaian establishments demonstrated better compliance in the partial fulfillment of requirements in cleanliness and staff hygiene behavior, with high levels of adherence observed in sanitary food preparation areas, shop layouts, and hygienic food handling practices. These indicators also showed relatively strong performance compared with other countries. Although overall compliance in Ghana was moderate, the pattern of mean responses indicates that basic hygiene behaviors are relatively well embedded in daily operations, whereas managerial and preventive control measures remain less developed [22]. This finding suggests that grocerants have achieved functional cleanliness and acceptable staff hygiene standards but still lack systematic oversight through documentation, monitoring, and pest‐management activities. Such partial implementation aligns with earlier findings from Ghana, where food handlers exhibited adequate food safety knowledge but failed to consistently apply it in practice [23]. Although Ghana′s food safety regulations are largely well established under the Public Health Act (“Act 851,” 2012) and the Food and Drugs Authority′s (FDA) mission, enforcement, and implementation remain challenging due to capacity and resource limitations [22].

Evaluation of the SUST data also revealed moderate compliance among Ghanaian grocerants. Sustainability initiatives tend to focus on economic and efficiency improvements, such as the use of energy‐efficient equipment or sustainable cleaning solutions. This pattern aligns with the *initial level* of sustainability maturity described by León Bravo et al., [24], where enterprises implement SUST beyond mandatory requirements to address stakeholder pressures like consumers and the community. However, assessments are often informal or nonstructured due to the company′s size and complexity. Companies focus on efficiency without developing a clear sustainability strategy. Consistent with the triple bottom line (TBL) perspective outlined by Bekele [25], sustainability assessment in food retail should encompass environmental, social, and economic dimensions. However, the absence of standardised sustainability reporting frameworks [25] constrains the ability of Ghanaian establishments to systematically document, evaluate, and communicate their progress toward sustainability goals.

The high compliance levels observed among Polish grocerants in both food safety and SUST indicate a mature operational culture, where regulatory discipline in food safety appears to be extending toward broader environmental and social responsibility. This strong performance can be attributed to the comprehensive, long‐standing harmonization of Polish food safety governance with the EU regulatory framework, which mandates the implementation of national systems like HACCP‐based systems and ensures rigorous oversight through coordinated inspection mechanisms [26, 27]. The findings of this study regarding the compliance level are in line with Rincon‐Ballesteros et al. [14], who affirm that developed countries have stable economic and social environments, ensuring food access and availability, responsible procurement and consumption habits, and strict legislation for safety. The findings are corroborated by Nyarugwe et al. [12], who reported that proactive food safety behavior and stronger compliance are typically found in well‐structured food governance systems and private standard participation. When compared with other countries, the developmental pattern aligns with the *committed level* described by León Bravo et al. [24], where retail organizations adopt a structured sustainability approach that extends beyond operational efficiency to include environmental and social considerations. At this level, companies demonstrate measurable progress and responsiveness to key stakeholders, particularly the community, reflecting a mature yet still evolving stage of sustainability integration.

Indian grocerants demonstrated a moderate level of compliance with FSP, reflecting partial institutionalization of hygiene systems within a rapidly evolving regulatory framework. Higher compliance was observed in preventive control approaches, including the separation of high‐risk foods, management of product expiry dates, prevention of contamination, and adherence to staff hygiene practices. These results indicate that the foundational principles of the Food Safety and Standards Authority of India (FSSAI) are being increasingly operationalised at the retail food service level. However, structural and managerial practices, such as facility layout, staff protective measures, and pest control measures, show weaker implementation. This uneven performance suggests that while basic hygiene behavior is embedded, systematic management of food safety remains inconsistent across grocerants, reflecting a transitional stage of FSMS adoption in India. Such findings mirror the national context, where despite the comprehensive Food Safety and Standards Act (2006), implementation remains uneven due to infrastructure gaps, limited inspection capacity, and the prevalence of unorganised food outlets [28, 29]. Qualitative research in Indian catering establishments also highlights comparable challenges, such as limited managerial commitment, insufficient infrastructure, and reliance on long‐standing informal practices that restrict the consistent implementation of food safety standards [30].

Adopting green practices can amplify environmental pressure on consumers and suppliers [31]. The findings in this study indicate that Indian grocerants have moderate efforts toward implementing sustainability approaches, which corresponds with India′s evolving sustainability governance structure, which has expanded considerably through initiatives under the Eat Right India framework [32]. Environmental approaches such as plastic reduction, reusable packaging, and local sourcing, where grocerants achieved higher compliance, mirror the diffusion of these national campaigns into everyday practices. The findings of this study can be supported by Bălan [33], who identified that retailers often act as key intermediaries in promoting sustainable consumption through marketing interventions, information cues, and product assortment. The observed sustainability pattern in Indian grocerants suggests that it can correspond to the *committed level* in sustainability [24]. However, lower performance in areas like food redistribution, waste segregation, and energy efficiency suggests that sustainability adoption remains selective and externally driven, focusing on visible environmental actions.

Three distinct clusters identified in this study represent distinct stages of food safety and sustainability maturity, shaped by differences in governance strength, operational capacity, and institutional culture. The lowest‐performing cluster exhibited characteristics of reactive systems, where hygiene and sustainability measures were inconsistently applied and driven mainly by individual effort. This mirrors findings from Nyarugwe et al., who reported reactive food safety culture in several African and Asian food companies due to weak enforcement and informal work structures [12]. Similarly, Rajic et al. [34] found that food businesses in non‐EU Eastern European countries displayed lower food safety climate maturity, which they linked to fragmented regulatory oversight. The intermediate cluster represented a transitional stage, characterised by a combination of partial compliance with selective SUST. This aligns with Rincón‐Ballesteros et al., who found that firms in Latin America often adopt food safety systems to meet commercial pressures rather than internalised food safety values, resulting in uneven FSMS performance [14]. The third cluster comprised mature systems, characterised by consistent implementation of both food safety and sustainability indicators. This pattern resembles high‐performing companies in Greece and Spain that exhibited proactive food safety culture and values–based FSMS motivation [12, 14]. Moreover, Spagnoli et al. provided preliminary empirical evidence that a more mature food safety culture in food processing firms is associated with increased investment in preventive and appraisal activities [21].

We also found a positive association between food safety management practices and SUST across the countries. Polish grocerants that demonstrate higher compliance with food safety standards also tend to implement SUST more effectively. This indicates a higher level of compliance in both domains, in which environmental responsibility is embedded within operational food safety management. Such compliance can be linked to the strong regulatory environment within the EU, where strict enforcement of food safety legislation is accompanied by increasing pressure to adopt sustainable production and retail practices. In Ghanaian grocerants, although the association is lower, it is nonetheless statistically significant, indicating an emerging alignment between safety and sustainability, even within existing infrastructural constraints.

The significant positive association between food safety and sustainability observed in Poland and Ghana aligns with the Green HACCP concept, which proposes the alignment of environmental and safety principles in food‐related operations [7]. However, the lack of a significant association in India highlights the fragmentation still present in several developing retail food systems. The observed relationship between food safety and SUST in grocerants directly supports achieving SDG 12.3, which targets a substantial reduction in food waste. As highlighted by Adams et al. [15], effective operational practices, such as demand forecasting and inventory management, are central to reducing waste and improving sustainability performance. These observations support the principles of green retailing, where sustainability is incorporated into daily operations rather than treated as a secondary or optional activity [31]. From a SDGs perspective, this integrated approach contributes directly to SDG 3 (good health and well‐being), SDG 12 (responsible consumption and production), and SDG 13 (climate action) by promoting a hygienic, safe, and environmentally responsible retail food environment.

Achieving food security and sustainable food production is fundamentally dependent on ensuring food safety [35]. Without implementing effective food safety measures, the goal of providing sufficient and secure food sources for the global population cannot be realized. As an initial exploration of grocerant compliance with food safety and sustainability requirements, this study comes with its limitations. One limitation is that the sample size may limit the generalizability of the findings; therefore, future studies should consider larger samples. Second, as a comparative assessment, this study covered only three nationalities, which may restrict broader generalization across different contexts. Finally, the study assessed compliance based on observable practices at the outlet level and did not account for broader contextual factors or include variables representing integrated operational practices linking food safety and sustainability. Future research should extend the analysis to additional countries to enhance the robustness of the findings. Moreover, future studies may adopt a systems‐based perspective that links food safety and sustainability outcomes across the entire food chain, thereby contributing to the development of more sustainable and resilient food systems.

## 5. Conclusion

The structured observation tool developed in this study enabled a cross‐sectional comparison of prevailing food safety and SUST among grocerants in Ghana (Africa), Poland (Europe), and India (Asia). The findings revealed clear maturity differences across countries: Ghanaian grocerants predominantly operated with reactive systems, Indian grocerants exhibited transitional compliance patterns, and Polish grocerants demonstrated a high level of maturity in food safety and SUST. Importantly, no grocerant fully met all indicators, underscoring that even in more advanced regulatory environments, gaps remain in achieving comprehensive compliance. The findings also showed that food safety and SUST are positively associated in contexts where management systems are more developed. This suggests that the convergence of food safety and sustainability remains an ongoing challenge globally, particularly in hybrid retail food service contexts. The findings supported the first hypothesis and partially supported the second, reflecting country‐level differences and a context‐specific association between food safety and SUST. The maturity framework applied here offers a practical tool for identifying progress and gaps and can support future benchmarking across regions or store types.

## Author Contributions

Surya Sasikumar Nair: conceptualization, methodology, formal analysis, data curation, investigation, writing—original draft, visualization, and writing—review & editing. Aparna Porumpathuparamban Murali: investigation. Richard Atinpoore Atuna: investigation and writing—review & editing. Fortune Akabanda: writing—review & editing and supervision. Wojciech Kolanowski: writing—review & editing. Shoukui He: visualization. Joanna Trafiałek: writing—review & editing, data curation, validation, and supervision.

## Funding

No funding was received for this manuscript.

## Ethics Statement

As this article does not contain any studies with human participants or animals performed by any of the authors, ethics approval was not required and the Declaration of Helsinki does not apply to this research.

## Conflicts of Interest

The authors declare no conflicts of interest.

## Data Availability

Data will be made available on request.
